# A case-control study on the combined effects of *p*53 and *p*73 polymorphisms on head and neck cancer risk in an Italian population

**DOI:** 10.1186/1471-2407-9-137

**Published:** 2009-05-08

**Authors:** Paola Gallì, Gabriella Cadoni, Mariangela Volante, Emma De Feo, Rosarita Amore, Arianna Giorgio, Dario Arzani, Gaetano Paludetti, Gualtiero Ricciardi, Stefania Boccia

**Affiliations:** 1 Institute of Hygiene Università Cattolica del Sacro Cuore, Rome, Italy; 2 Institute of Otorhinolaryngology, Università Cattolica del Sacro Cuore, Rome, Italy

## Abstract

**Background:**

The purpose of this study is to analyze the combined effects of selected *p*53 and *p*73 polymorphisms and their interaction with lifestyle habits on squamous cell carcinoma of the head and neck (SCCHN) risk and progression in an Italian population.

**Methods:**

Two hundred and eighty-three cases and 295 hospital controls were genotyped for *p*53 polymorphisms on exon 4 (Arg72Pro), intron 3 and 6, and *p*73 G4C14-to-A4T14. Their association with SCCHN was estimated using a logistic regression analysis, while a multinomial logistic regression approach was applied to calculate the effect of the selected polymorphisms on SCCHN different sites (oral cavity, oropharynx, hypopharynx and larynx). We performed an haplotype analysis of the *p*53 polymorphisms, and a gene-gene interaction analysis for the combined effects of *p*73 G4C14-to-A4T14 and *p*53 polymorphisms.

**Results:**

We found a significant increased risk of SCCHN among individuals with combined *p*73 exon 2 G4A and *p*53 intron 3 variant alleles (OR = 2.22, 95% CI: 1.08–4.56), and a protective effect for those carrying the *p*53 exon 4-*p*53 intron 6 diplotype combination (OR = 0.67; 95% CI: 0.47–0.92). From the gene-environment interaction analysis we found that individuals aged < 45 years carrying *p*73 exon 2 G4A variant allele have a 12.85-increased risk of SCCHN (95% CI: 2.10–78.74) compared with persons of the same age with the homozygous wild type genotype. Improved survival rate was observed among *p*53 intron 6 variant allele carriers (Hazard Ratio = 0.51 (95% CI: 0.23–1.16).

**Conclusion:**

Our study provides for the first time evidence that individuals carrying *p*53 exon 4 and *p*53 intron 6 variant alleles are significantly protected against SCCHN, and also shows that an additional risk is conferred by the combination of *p*73 exon 2 G4C14-to-A4T14 and *p*53 intron 3 variant allele. Larger studies are required to confirm these findings.

## Background

Squamous cell carcinoma of the head and neck (SCCHN), including cancers of the oral cavity, pharynx and larynx, is one of the five most common cancers worldwide [1]. Epidemiological studies suggest a multifactorial aetiology, with smoking, alcohol drinking [2,3], oral HPV infection [4], low fruit and vegetables intake [5,6], and genetic factors playing a major role [7].

The *p*53 protein, encoded by *p*53 tumor suppressor gene (17p13), is involved in cell-cycle regulation, DNA repair, inhibition of spontaneous mutations, and cellular differentiation and apoptosis [8]. The *p*53 gene is highly polymorphic, with at least 13 different polymorphisms described [9,10]. Some studies reported that rare *p*53 exon 4 – intron 3 – intron 6 haplotypes affect the frequency of mutation and loss of heterozygosity in B-lymphoblastoid cell lines, along with DNA repair and apoptosis [11,12]. Currently *p*53 exon 4 Arg72Pro polymorphism is the only *p*53 single nucleotide polymorphism (SNP) whose effect has been studied in relation to SCCHN, with conflicting results. Three studies conducted on Caucasians [13-15] reported the absence of a significant relationship between Pro variant allele and SCCHN, while Tsai et al [16] suggested an increased cancer risk for individuals carrying the variant allele in a Chinese population. Two intronic polymorphisms of *p*53 consisting of a 16-bp duplication in intron 3 and a G-A transition in intron 6, have been investigated in relation to lung, ovarian, breast and colon cancer, again with conflicting results [12,17-19]. To our knowledge, no one study thus far has investigated the effect of these two SNPs on SCCHN.

The *p*73 protein is a *p*53 homologue encoded by a polymorphic gene located on 1p36-33, mapping on a region often deleted in a variety of human cancers [20,21]. *p*73 protein activates several *p*53-responsive genes involved in cell cycle control, DNA repair and apoptosis, and inhibits cell growth in a *p*53-like manner by inducing apoptosis [22]. A *p*73 G4C14-to-A4T14 dinucleotide polymorphism has been reported to influence gene expression, and consists of two SNPs in complete linkage disequilibrium at positions 4 (G-A) and 14 (C-T) in the non-coding region of exon 2 [23]. Several studies investigated the relationship between the *p*73 G4C14-to-A4T14 polymorphism and cancer [10,24-27], among them the large study conducted by Li et al. reported an increased risk for SCCHN in Caucasians carrying the *p*73 G4C14-to-A4T14 variant genotype compared to GC/GC [10].

To our knowledge, no one study investigated the influence of *p53 *intron 3 and 6 polymorphisms on SCCHN survival, while a poor disease-free survival has been reported in patients carrying the *p*53 exon4 Arg72Pro variant on SCCHN, lung, colorectal and breast cancer [28-31]. Lastly, a study reports an improved survival of colorectal cancer patients carrying the variant allele of *p*73 G4C14-to-A4T14 [32].

The purpose of our study was to investigate the effects of *p*73 G4C14-to-A4T14 polymorphism and three *p*53-SNPs in exon 4 and introns 3 and 6, as well as their combination and their interaction with lifestyle habits, in association with SCCHN risk and progression within a hospital-based case-control study in an Italian population.

## Methods

### Study Population

The study subjects were added to the age and gender matched sample of the previously published case-control study [5]. Study participants were recruited from among patients admitted to the teaching hospital "A. Gemelli" of the Università Cattolica del Sacro Cuore (Rome, Italy) during the time period from May 2002 to May 2007, and eligibility was restricted to Caucasian individuals born in Italy. Cases were consecutive primary untreated head and neck cancer patients admitted to the Department of Otorhinolaryngology with histologically-confirmed squamous cell carcinoma of the head and neck (SCCHN). SCCHN diagnosis was defined according to the International Classification of Disease (9^th ^revision, codes 140–149 and 161). Tumors were staged according to the UICC-TNM classification; 48.9% were staged I-II and 53.9% were classified T1-T2 (tumor stage was missing for one patient, and tumor grade was missing for one patient) [33]. Cases presented the following distribution according to tumor site: 67.7% laryngeal, 18.6% oral cavity, 6.5% oropharyngeal, and 8.2% hypopharyngeal. Controls were selected from among cancer-free patients admitted to the same hospital during the same time period with a broad range of diagnoses without matching to cases by any covariate. The study sample size comprised 283 cases and 295 controls, with a participation rate of 98% among cases and 93% among controls. Written informed consent was obtained from all study subjects, after which each subject provided a venous blood sample that was collected into EDTA-coated tubes. This study was performed according to the Declaration of Helsinki and was approved by the ethics committee of the Università Cattolica del Sacro Cuore.

### Genotyping

Genotyping for *p*53 intron 3 (rs17883323), *p*53 exon 4 codon 72 (rs1042522) and *p*53 intron 6 (rs1625895) polymorphisms were performed using a polymerase chain reaction (PCR) followed by restriction-fragment length polymorphism, as already described by Wu et al [12]. Genotyping of *p*73 exon 2 (rs2273953-rs1801173) was determined using DNA amplification, conducted as described by Niwa et al [25]. We conducted haplotype analysis for *p*53 intron 3, *p*53 exon 4 codon 72 and *p*53 intron 6 polymorphisms using EH software [34] and cocaphase [35]. Quality control for each genotyping was performed in each experiment, and 10% of the total samples were randomly selected and retested with 100% concordance. The analyst was blinded to the case or control status of the samples.

### Data Collection

A standard questionnaire was administered to cases and controls by trained medical doctors to elicit information on demographic variables, including cigarette smoking, drinking history, fruit and vegetable intake, physical activity, family history of cancer, and family history of SCCHN specifically. Participants were asked to focus on the year prior to diagnosis (for controls the year prior to the interview date) when answering questions regarding lifestyle. Pack-years were calculated as years smoked multiplied by the current number (or previous number, for those who had quit) of cigarettes smoked/day divided by 20. For quantification of fruit and vegetable intake, 70 grams was considered an average portion of vegetables (both fresh and cooked vegetables included) and 100 grams was considered an average portion of fruit [36]. Participants were classified as consuming ≥ 2 portions/day if they regularly consumed at least one portion of fruit and one portion of vegetables per day or if they consumed at least 2 portions of fruit or 2 portions of vegetables per day. Cases were followed-up after histopathological diagnosis of cancer with a median follow-up time of 23 months (range 1–60 months), and information on recurrence of local and/or regional neck nodes was also recorded. Data concerning previous Human Papilloma Virus (HPV) infection were not available for either cases or controls as well as treatment practices adopted by cases.

The proportion lost to follow-up among cases in the study cohort was 6.0%. The response rate for interview completion was 98.0% among cases and 96.0% among controls, with the exception of data relating to the family history of cancer (unknown in 22% of cases and 3.4% of controls), as well as the family history of oral cancer (unknown in 28.7% of cases and 3.4% of controls).

### Statistical analysis

The relationship between SCCHN and putative risk factors were measured using the adjusted odds ratios (ORs) and their 95% confidence intervals (CI) derived from logistic regression analysis using STATA software (version 9). The SCCHN risk according to the 4 studied polymorphisms was estimated under the dominant model (variant allele carriers *versus *wild type allele homozygous). We considered possible risk factors for SCCHN as potential confounders if addition of that variable to the model changed the OR by 10% or more. Confounding checks were performed in both univariate and final multivariate models. If a factor was identified as a confounder of any estimated main effect, it was kept in all models. Based on these criteria, we controlled for gender, alcohol, pack-years of cigarette smoking, physical activity and fruit and vegetable intake. With exception of gender, we adjusted for confounders as continuous variables. After a logistic regression model has been fitted, the Hosmer-Lemeshow test was performed to assess the goodness of the model.

We tested the relationship between the analyzed polymorphisms and tumour location (oral cavity, oropharynx, hypopharynx and larynx cancer) [3] by using a logistic multinomial regression analysis, using healthy controls as the reference group.

The False Discovery Rate (FDR), which controls the expected proportion of false positives among a threshold given by the observed *p*-value distribution [37], was performed to correct for multiple comparisons resulting from association between the four polymorphisms and the different tumour locations.

Tests of Hardy Weinberg Equilibrium (HWE) were carried out for all of the polymorphisms among cases and controls separately http://ihg.gsf.de/cgi-bin/hw/hwa1.pl. In order to examine if the effects of the studied polymorphisms are modified by selected environmental exposures and some other covariates, we performed a logistic regression analysis stratified by age, gender, alcohol, smoking status and family history of cancer. An homogeneity test was then used to test differences among the strata. In this analysis we used as a reference group those homozygous wild-type individuals who had not been exposed and confounders were considered as follows: age was categorized into ≤ 45 and > 45 years old [38], smoking status was considered as ever/never cigarette smokers, and alcohol consumption as drinkers/non-drinkers (the latter including individuals whose alcohol intake was less than 7 g/day). A gene-gene interaction analysis was performed among *p*73 G4C14-to-A4T14 polymorphism and three *p*53-SNPs in exon 4 and introns 3 and 6, and the likelihood ratio test was used to test for more than multiplicative effect among each pair of SNPs, with the homozygous wild-type individuals used as the reference group for each gene.

Overall survival curves were calculated by the Kaplan-Meier product limit method from the date of diagnosis until progression of disease or death. If a patient was not dead, survival was censored at the time of the last visit. The log rank test was used to assess differences between subgroups. Disease Free Survival (DFS) was calculated from the day of histological diagnosis to the date of local recurrence of disease and/or regional lymph-node involvement. The risk of death and the risk of recurrence related to the studied polymorphisms were estimated by Cox's proportional hazards model. Hazard ratios (HR) were adjusted for age, gender, and stadium, with the wild-type genotypes as the reference group.

## Results

General characteristics of the study population are presented in Table 1. The percentage of males was higher in cases then in controls (82.7% vs 60.0%; Table 1). Results show that alcohol consumption increases head and neck cancer risk, especially among heavy drinkers (OR = 18.56; 95% CI: 7.26–47.44). An increased risk was also detected for cigarette smokers, with an OR of 2.97 (95% CI: 1.72–5.12) and 8.23 (95% CI: 4.73–14.32) for 1–24 and > 24 pack-years of cigarettes smoked, respectively (Table 1). A low consumption of fruit and vegetables (< 2 portions/day) was significantly associated with SCCHN (OR = 2.70; 95% CI: 1.73–4.21). Additionally, family history of cancer was higher in cases than controls (OR = 2.42; 95% CI: 1.42–4.13), and even higher when considering the family history of SCCHN, although this is limited to a very small numbers of individuals (data not shown). Goodness of fit statistics provided a *p*-value > 0.42.

**Table 1:** Odds Ratios (95% Confidence Interval) for squamous cell carcinoma of the head and neck (SCCHN) according to the collected variables.

	283 Cases	295 Controls	OR° (95% CI)
	n (%)	n (%)	
Age (years ± SD)	63.3 ± 11.2	63.5 ± 13.1	-
Male gender	234 (82.7)	177 (60.0)	0.85 (0.50 – 1.42)
Alcohol drinkers			
0 g/day	59 (20.9)	146 (49.7)	1*
1–30 g/day	119 (42.2)	141 (48.0)	1.58 (0.98 – 2.52)
> 31 g/day	104 (36.88)	7 (2.38)	18.56 (7.26 – 47.44)
Pack-years of smoking			
0	58 (18.5)	179 (62.0)	1*
1–24	67 (23.8)	66 (22.8)	2.97 (1.72 – 5.12)
> 24	162 (57.7)	44 (15.2)	8.23 (4.73 – 14.32)
Fruit and vegetables intake			
≥ 2 portions/day	116 (41.1)	198 (69.5)	1*
< 2 portion/day	166 (58.9)	87 (30.5)	2.70 (1.73 – 4.21)
Physical activity			
≥ 2 times/week	15 (5.5)	30 (10.2)	1*
< 2 time/week	259 (94.5)	263 (89.8)	1.56 (0.68 – 3.57)
Family history of cancer			
No	137 (62.0)	232 (81.4)	1*
Yes	84 (38.0)	53 (18.6)	2.42 (1.42 – 4.13)

* Reference group

° Odds Ratio (OR) adjusted for gender, alcohol, pack-years, physical activity and fruit and vegetables intake

The genotype frequencies of our control group were in line with those of Caucasians [39,40] and were in HWE for both cases and controls (*p*- value > 0.05). As shown in Table 2, the distribution of the studied polymorphisms was similar amongst the two groups. A significant association was observed between *p*73 variant allele carriers and cancer of the oral cavity (OR = 2.43; 95% CI: 1.23–4.81; *p*-value = 0.01), even if it didn't remain noteworthy after the FDR test correction because the statistically significant threshold was set at *p *= 0.0002. The estimated *p*53 pairwise haplotype frequencies among the three polymorphisms and their linkage disequilibrium values in cases and controls are shown in Table 3. The pairwise linkage disequilibria were highly significant in both control and patient population (*p *value for χ^2 ^test < 0.001). The frequency of exon 4 (Pro)/intron 6 diplotype was lower in cases than in controls (12.1% versus 17.6%), with more than 30% reduced risk of SCCHN (OR = 0.67; 95% CI: 0.47–0.92). There were no significant differences in the remaining diplotype frequencies among cases and controls (*p *value > 0.1, Table 3). When the estimation of haplotype frequencies was extended to all the three SNPs (Table 4), results showed no significant difference in the haplotype frequencies among cancer patients and controls (*p *value > 0.1). From the *p*73-*p*53 gene-gene interaction analysis a more than additive effect was found for those carrying both *p*53 intron 3 and *p*73 variant alleles, with an OR of 2.22 (95% CI: 1.08–4.56) (Table 5) [41].

**Table 2:** Odds Ratios (95% CI) for head and neck cancer according to the studied polymorphisms

	283 Cases	295 Controls	OR °	OR° for oral cavity (52 cases)	OR° for oropharynx (18 cases)	OR° for hypopharynx (23 cases)	OR° for larynx (186 cases)
	N (%)	N (%)	*(95% CI)*	*(95% CI)*	*(95% CI)*	*(95% CI)*	*(95% CI)*
*p*53 intron 3 (rs1788332)							
WW	191 (67.5)	209 (70.8)	1*	1*	1*	1*	1*
M carriers	92 (32.5)	86 (29.2)	1.30 (0.81 – 2.10)	1.46 (1.78 – 2.88)	1.75 (0.61 – 5.00)	0.55 (0.18 – 1.75)	1.17 (0.69 – 1.98)
*p*53exon4Arg72Pro (rs1042522)							
ArgArg	175 (61.85)	169 (57.3)	1*	1*	1*	1*	1*
Pro carriers	108 (38.2)	126 (42.7)	1.07 (0.68 – 1.67)	1.02 (0.53 – 1.97)	1.66 (0.59 – 4.62)	0.87 (0.34 – 2.22)	0.91 (0.55 – 1.49)
*p*53 intron 6 (rs1625895)							
GG	194 (68.5)	192 (65.1)	1*	1*	1*	1*	1*
C carriers	89 (31.5)	86 (34.9)	0.99 (0.63 – 1.58)	0.49 (0.23 – 1.05)	1.28 (0.45 – 3.65)	0.70 (0.26 – 1.91)	1.00 (0.60 – 1.67)
*p*73 exon 2 G4A (rs1801173/rs2273953)							
GC/GC	187 (66.1)	214 (72.5)	1*	1*	1*	1*	1*
AT carriers	96 (33.9)	81 (27.5)	1.51 (0.94 – 2.43)	2.43 (1.23 – 4.81)^§^	1.99 (0.69 – 5.69)	0.48 (0.13 – 1.71)	1.46 (0.86 – 2.46)

* Reference group

° OR adjusted for gender, alcohol, pack-years, physical activity and fruit and vegetables intake

^§ ^This association (p-value = 0.01) didn't remain noteworthy after the FDR test correction because the statistically significant threshold was set at *p*-value = 0.0002.

**Table 3:** Pairwise haplotype frequencies in SCCHN patients and controls.

	No. alleles	Estimated haplotype frequency				D'
		‡ 1 - 1	1 - 2^	2 - 1	2 - 2	
Intron 3-exon 4 †						
Cases	566	0.740	0.052	0.086	0.120	0.61
Controls	590	0.716	0.030	0.120	0.131	0.74

Intron 3-intron 6†						
Cases	566	0.772	0.055	0.058	0.115	0.61
Controls	590	0.763	0.074	0.035	0.128	0.73

Exon 4-intron 6*						
Cases	566	0.744	0.049	0.086	0.121	0.64
Controls	590	0.719	0.026	0.076	0.176	0.83

† *p*-value of χ^2^-test: cases versus controls > 0.1

* *p*-value < 0.001

‡ 1 = intron 3 (W), exon 4 Arg, intron 6 G

^ 2 = intron (M), exon 4 Pro, intron 6 A

**Table 4:** Estimated *p*53 haplotype frequencies in SCCHN patients and controls.

	Alleles	‡1 - 1 - 1	1 - 1 - 2^	1 - 2 - 1	1 - 2 - 2	2 - 1 - 1	2 - 1 - 2	2 - 2 - 1	2 - 2 - 2
Intr 3-ex 4-intr 6									
Cases	566	0.705	0.034	0.067	0.120	0.040	0.014	0.019	0.101
Controls	590	0.696	0.020	0.068	0.053	0.026	0.005	0.008	0.123

*p*-value of χ^2^-test: cases versus controls > 0.1

‡ 1 = intron 3 (W), exon 4 Arg, intron 6 G

^ 2 = intron (M), exon 4 Pro, intron 6 A

**Table 5:** Adjusted Odds Ratios (95% CI) of SCCHN for the *p*73 exon 2 G4A- *p*53 interaction analysis.

		*p*53 intron 3 (rs1788332)		*p*53 intron 6 (rs1625895)		*p*53 exon4 Arg72Pro (rs1042522)	
		wt/wt	mt carriers	wt/wt	mt carriers	wt/wt	mt carriers
	wt/wt	1*	1.05 (0.57–1.91)	1*	0.94 (0.53–1.68)	1*	0.92 (0.53–1.61)
	cases/controls	136/152	51/62	137/140	50/74	129/124	58/90
*p*73 exon 2 G4A	mt carriers	1.25 (0.70–2.25)	2.22 (1.08–4.56)	1.49 (0.82–2.70)	1.50 (0.75–3.02)	1.33 (0.69–2.55)	1.63 (0.86–3.11)
(rs1801173/rs2273953)	cases/controls	56/56	40/24	57/51	39/29	45/44	51/36
	
		*p *for interaction† = 0.30		*p *for interaction† = 0.89		*p *for interaction† = 0.55	

* = Reference category

† = By likelihood ratio test

From our analysis there was no evidence of effect modification of the four SNPs by the environmental exposures (data not shown). Age was an effect modifier of the association between *p*73 exon 2 and SCCHN [OR = 12.85 (95% CI: 2.10–78.74) in individuals younger than 45 years *versus *an OR = 1.19 (95% CI: 0.72–1.96) in individuals older than 45 years (*p *value of homogeneity among the estimates = 0.013)]. In addition, current smokers carrying the variant allele of *p*73 exon 2 had an OR of 3.60 (95% CI: 1.30–9.92) for SCCHN, while never smokers an OR of 1.32 (95% CI: 0.80 – 2.19; *p *value of heterogeneity among strata estimates = 0.10). Lastly, female smokers carrying the variant allele of *p*73 exon 2 had an OR of 4.18 (95% CI: 0.92–19.02) for SCCHN versus an OR of 1.07 (95% CI: 0.54–2.14) among smoker males (*p *value of homogeneity = 0.10).

**Figure 1:**
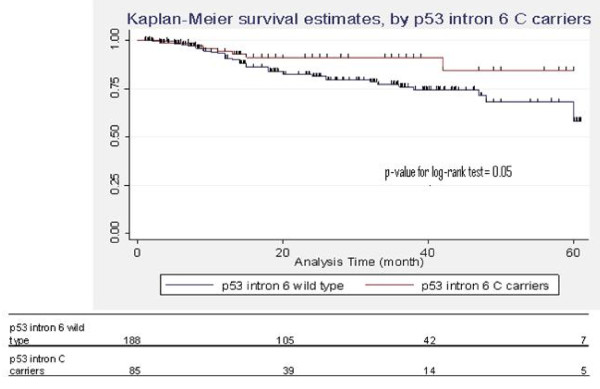
**Association between *p *53 intron 6 and overall survival among 283 SCCHN cases**.

Overall actuarial 2-year survival rate was 84.4% (95% CI: 79.7–88.2) with a 2-year DFS rate of 81.2% (95% CI: 76.2–85.3). During the follow-up period local recurrence was observed in 53 cases, among them five patients also experienced metastatic regional lymph-node involvement. The survival analysis showed no significant association between any of the polymorphisms, overall survival and DFS (data not shown), even though individuals carrying *p*53 intron 6 variant allele had an HR for the overall survival of 0.51 (95% CI: 0.23–1.16) (*p*-value for log-rank test = 0.05) (Figure 1).

## Discussion

This case-control study evaluated the effect of four polymorphisms in the *p*53 and *p*73 genes on the risk of squamous cell carcinoma of the head and neck in an Italian population. To our knowledge, our's is the first study reporting an increased risk of SCCHN among individuals with combined *p*73 exon 2 G4A and *p*53 intron 6 variant alleles and a protective effect for those carrying *p*53 exon 4- *p*53 intron 6 diplotype combination. Additionally, we showed that *p*73 exon 2 G4A variant allele increases the risk of cancer of the oral cavity, which is in line with the results of Li et al [10]. From the gene-environment interaction analysis we showed that individuals aged < 45 years carrying *p*73 exon 2 G4A variant allele have 12.85-times significant increased risk for SCCHN compared with persons of the same age with the homozygous wild type genotype. Even though this observation stems from a subgroup made up of less than 100 individuals, it confirms the results of Li et al [10]. The absence of an association between *p*53 exon 4 and SCCHN confirms the results of three published studies among Caucasians [13-15], while the absence of a significant association between *p*53 introns 3 and 6 and SCCHN is in line with the results of other tumour sites [42,43]. Lastly, carriers of the variant allele of *p*53 intron 6 showed a poorer prognosis following cancer diagnosis.

In interpretation of our results some limitations of the study should be taken into account. Firstly, given the results of our *a posterior *power calculation on the basis of the prevalence of the analyzed polymorphisms in our control population, our study is powered to detect a minimum OR of 2.0 for the effect of variant allele carriers for all studied polymorphisms (with a significance level of 5%). The study's sample size limits the possibility to explore the combined effects of the genotypes, or gene-environment interactions, thus we need to increase the sample size in order to confirm our results. However, when appropriately conducted, large and small studies should theoretically give the same results, but with a more precise effect measure estimate in the larger ones [44]. Secondly, as in all case-control studies, information bias may exist, leading to biased ORs of the effects of lifestyle and environmental exposures, alone or in combination with a gene polymorphism. Thirdly, data on HPV infection were unavailable in our study population.

Our study reports for the first time an increased risk of SCCHN in individuals carrying both *p*73 exon 2 and *p*53 intron 3 unfavourable variants. Taking into account all the possible combinations of *p*73 exon 2 and *p*53 intron 3 alleles, the large study Schabath et al. [22] recently reported a 13% increased risk of lung cancer for individuals carrying every additional variant allele, however they did not differentiate among variant alleles. We did not report any effect for the remaining *p*73-*p*53 gene-gene interactions, thus confirming the results from Niwa et al on *p*73 exon 2-*p*53 exon 2 and endometrial cancer [25]. The results of the *p*53 haplotype analysis and SCCHN show that individuals carrying combined unfavourable variants of *p*53 exon 4 and intron 6 are protected from SCCHN, and these results are in line with those reported for lung, breast and gastric cancer [12,45,46]. Due the potential application of these results in cancer prevention, additional *in vitro *studies are urgently required to clarify the mechanism through which the combined effect of these strongly linked *p*53 SNPs might influence the protein function.

Our study shows that *p73 *exon 2 G4C14-to-A4T14 polymorphism increases the risk of cancer of the oral cavity, even though the result is no longer significant after correction for multiple testing. This is in line, however, with results from Li et al [10], and for other tumour sites such as colorectal, oesophageal, and gastric cancer among Caucasians [10,24,32]. Additionally, *p73 *exon 2 G4C14-to-A4T14 polymorphism seems to increase the risk of SCCHN particularly among individuals younger than 45, where the genetic susceptibility usually plays a major role in the early onset of SCCHN with respect to the environmental exposures [24]. Lastly, gender appears to be an effect modifier of the association between *p*73 AT variant allele and SCCHN, with female smokers at an increased risk compared to males, as reported from Li et al. [10]. Based on previous findings showing that women tend to have a lower capacity of DNA repair with respect to men [26], it is possible that women carrying the *p*73 G4C14-to-A4T14 variant allele are more sensitive to carcinogens. In order to confirm the potential role of the *p73 *G4C14-to-A4T14 polymorphism on SCCHN, however, further studies on its functional effect are needed. Since this polymorphic site is located in a non coding region it remains to be explained how the variant allele can modulate the protein function. Also, it should be verified that *p73 *G4C14-to-A4T14 does not exists in linkage disequilibrium with functional alleles at other susceptibility loci [24].

The results of the present study shows a decreased mortality rate for individuals carrying the variant allele of *p*53 intron 6 compared with the wild-type genotype. A similar result has been reported for the intestinal histotype of gastric cancer (De Feo et al. A case-control study on the effect of p53 and p73 polymorphisms on gastric cancer risk and progression in an Italian population. 'Mutation Research, Genetic Toxicology and Environmental Mutagenesis', in press) and chronic lymphocitic leukaemia patients when considering the time of treatment-free survival as the main outcome [45]. Due to the small number of people involved in the survival anlaysis, however, further research should confirm our preliminary evidence.

## Conclusion

Our study provides for the first time evidence that individuals carrying both *p*53 exon 4 and *p*53 intron 6 variant allels are significantly protected against SCCHN, while an additional risk is conferred by the combination of *p*73 exon 2 G4C14-to-A4T14 and *p*53 intron 3 variant alleles. We confirm the existing evidence that *p*73 exon 2 G4C14-to-A4T14 increases the risk of oral cavity cancer. Larger prospective studies are needed to further confirm our results.

## Abbreviations

CI: Confidence Interval; DFS: Disease Free Survival; HR: Hazard Ratio; HWE: Hardy-Weinberg Equilibrium; OR: Odds Ratio; PCR: Polymerase Chain Reaction; SCCHN: Squamous Cell Carcinoma of the Head and Neck; SNP: Single Nucleotide Polymorphism.; FDR: False Discovery Rate.

## Competing interests

The authors declare that they have no competing interests.

## Authors' contributions

SB and GR conceived the study and coordinated the research group; PG, EDF and SB performed the statistical analysis and drafted the manuscript; DA and RA carried out the genotyping; GC, MV, AG and GP participated in the study design, enrolled the patients and interviewed them.

## Pre-publication history

The pre-publication history for this paper can be accessed here:

http://www.biomedcentral.com/1471-2407/9/137/prepub
